# Superior Non-Invasive Glucose Sensor Using Bimetallic CuNi Nanospecies Coated Mesoporous Carbon

**DOI:** 10.3390/bios11110463

**Published:** 2021-11-18

**Authors:** Ahmed Bahgat Radwan, Sreedevi Paramparambath, John-John Cabibihan, Abdulaziz Khalid Al-Ali, Peter Kasak, Rana A. Shakoor, Rayaz A. Malik, Said A. Mansour, Kishor Kumar Sadasivuni

**Affiliations:** 1 Center for Advanced Materials (CAM), Qatar University, Doha P.O. Box 2713, Qatar; sreedevisathyan96@gmail.com (S.P.); peter.kasak@qu.edu.qa (P.K.); shakoor@qu.edu.qa (R.A.S.); 2 Department of Mechanical and Industrial Engineering, Qatar University, Doha P.O. Box 2713, Qatar; john.cabibihan@qu.edu.qa; 3 Department of Computer Engineering, Qatar University, Doha P.O. Box 2713, Qatar; a.alali@qu.edu.qa; 4 KINDI Center for Computing Research, Qatar University, Doha, P.O. Box 2713, Qatar; 5 Weill Cornell Medicine-Qatar, Qatar Foundation-Education City, Doha P.O. Box 24144, Qatar; ram2045@qatarmed.cornell.edu; 6 Qatar Energy and Environment Research Institute, Hamad bin Khalifa University, Qatar Foundation, Doha P.O. Box 34110, Qatar; smansour@hbku.edu.qa

**Keywords:** sweat sensor, glucose detection, non-invasive, mesoporous carbon, bimetallic nanomaterials

## Abstract

The assessment of blood glucose levels is necessary for the diagnosis and management of diabetes. The accurate quantification of serum or plasma glucose relies on enzymatic and nonenzymatic methods utilizing electrochemical biosensors. Current research efforts are focused on enhancing the non-invasive detection of glucose in sweat with accuracy, high sensitivity, and stability. In this work, nanostructured mesoporous carbon coupled with glucose oxidase (GO_x_) increased the direct electron transfer to the electrode surface. A mixed alloy of CuNi nanoparticle-coated mesoporous carbon (CuNi-MC) was synthesized using a hydrothermal process followed by annealing at 700 °C under the flow of argon gas. The prepared catalyst’s crystal structure and morphology were explored using X-ray diffraction and high-resolution transmission electron microscopy. The electrocatalytic activity of the as-prepared catalyst was investigated using cyclic voltammetry (CV) and amperometry. The findings show an excellent response time of 4 s and linear range detection from 0.005 to 0.45 mM with a high electrode sensitivity of 11.7 ± 0.061 mA mM cm^−2^ in a selective medium.

## 1. Introduction

The accurate quantification of glucose is key to the diagnosis and management of patients with diabetes. In 2019, the International Diabetes Federation estimated that diabetes affects 463 million people, constituting 9.3% of the adult population aged 20–79, but with 1 in 2 remaining undiagnosed. Chronic hyperglycemia is a major risk parameter for long-term microvascular and macrovascular complications leading to increased morbidity and mortality, with 11.3% of global deaths being attributed to diabetes [1,2]. Additionally, in patients with COVID-19, whilst long-term glycemic control has not been associated with adverse outcomes, the severity of hyperglycemia at admission has been allied to the prerequisite for mechanical ventilation, admission to the intensive care unit, and mortality, independent of age, diabetes, and hypertension [3,4].

There is increasing use of bioelectrode interfaces with wearable bioelectronics to enable the continuous non-invasive transfer of physiological information [5,6,7,8,9,10]. In addition, the detection of biochemical molecules to indicate physiology and metabolism can be achieved using wearable electrochemical biosensors [11,12,13]. These electrochemical bio-sensors are generally established on enzyme–electrochemical reactions with high specificity [14]. The key step for wearable electrochemical biosensors is the enzyme–electrode interface where the electron transfer takes (ET) place [15,16,17]. The ET between enzymes and electrodes is problematic, as the polypeptides are embedded with the enzymes at the redox-active center [18,19]. Accordingly, to overcome this issue, electron mediators have been used to enhance the enzyme–electrochemical reactions. However, cross-reaction and leakage of the electron mediators result in low selectivity and instability of the biosensors [20,21]. A wearable electrochemical biosensor with high sensitivity and selectivity for biochemical molecules requires direct ET at enzyme–electrode interfaces without electron mediators. For a wearable sensor, a sensing platform is also essential for reproducibility, real-time detection, and high sensitivity. In 2014, Google partnered with Novartis to develop a smart contact for real-time assessment of tear glucose levels but was subsequently abandoned as there was a poor correlation between blood glucose levels and tears.

Sensing glucose in sweat is easy and avoids penetrating the skin to access blood or interstitial fluid. The excretion of glucose in sweat primarily depends on blood glucose; for a healthy individual, the sweat glucose level is 0.02–0.6 mM and 0.01–1 mM for diabetic patients [22,23,24,25,26]. It has been suggested that the electrochemical analysis of glucose concentration in sweat could accurately reflect blood glucose levels [27,28]. Although a recent study showed no correlation between sweat and blood glucose concentrations after submaximal exercise, indicating that sweat composition is at least relatively independent of blood composition [29].

Enzymatic sensors relying on glucose oxidation have been developed to detect glucose from sweat with high sensitivity but have a short lifespan of 1–2 weeks [30]. Interfaces of biological components are not necessary for nonenzymatic glucose sensors, and, therefore, they are cheaper to fabricate and have a longer life span [31]. However, they are less efficient because of their high working potential, slow kinetics, and weak sensing parameters with poor selectivity and low sensitivity [32]. An alternative metal and metal oxide can be used at the interface of nanoporous gold to overcome some of these issues [33,34], but this is expensive and limits commercial usage since the fabrication of low-cost glucose sensors is a market prerequisite [35]. Prussian blue/hydrogen peroxide has been utilized to enhance glucose sensitivity and selectivity in sweat, but it lacks stability and selectivity. Nanomaterials have also shown excellent performance in sweat-based electrochemical glucose biosensing systems. However, nanomaterials such as ZnO nanowires, Cu NCs, CNTs cannot be used efficiently because of their high working potential, pH conditions, low kinetics, and sensing parameters [36]. Therefore, researchers have integrated nanomaterial having high electroconductive and large surface area with glucose oxidase (GOx) enzymes to enhance selectivity and sensitivity. It is worth mentioning that one research group synthesized hierarchical mesoporous CNTs to achieve a sensitivity of 270 ± 10 μA mM^−1^ cm^−2^. CuNi glucose-sensing includes good reproducibility, reusability, and stability given the controllable preparation of electrodes and the surface with a robust chemical state. The disadvantage is that glucose-sensing can only be achieved at higher pH.

Glucose levels have primarily been evaluated from blood samples, although there is now increasing use of continuous glucose monitoring, relying on subcutaneous interstitial glucose levels [37,38,39]. This is particularly relevant to assess hypoglycemia, which can occur during Ramadan due to glucose-lowering therapies, particularly insulin [40,41]. Other body fluids such as sweat, saliva, and tears may also be utilized to assess glucose levels; however, the glucose amount in these body fluids is very low, and the sensing devices require high sensitivity to detect glucose. In this context, biosensors have high sensitivity and better stability than traditional methods of glucose detection [42]. Non-invasive glucose monitoring helps in continuous glucose monitoring by providing real-time and dynamic information about the glucose concentration. This method of glucose monitoring measures glucose without puncturing the skin to withdraw blood and without causing pain or trauma [43].

In this work, we have developed non-invasive glucose biosensing based on a Ni-Cu/mesoporous carbon/GOx nanocomposite. The synthesis method is cost-effective and straightforward without bulky and expensive instruments. The prepared composite was analyzed using XRD and TEM, and cyclic voltammetry was performed at various glucose concentrations in sweat samples. In addition, chronoamperometry was used to evaluate the sensitivity and interference (fructose, sucrose, uric acid, and dopamine) performance of the electrode in artificial sweat samples for glucose detection at pH 12.

## 2. Experimental

### 2.1. Materials

Sodium hydroxide was purchased from Scharlau, glucose and sucrose were purchased from Fisher scientific. Uric acid and fructose were bought from the Research lab; however, NaCl was purchased from Avonchem. Urea and lactic acid were bought from Alfa Aesar. Mesoporous carbon, copper acetate Cu(CH_3_COO)_2_·3H_2_O and nickel acetate Ni(CH_3_COO)_2_·6H_2_O, N, N-dimethylformamide (DMF) oleic acid, potassium chloride KCl, dopamine, and oleylamine were purchased from Sigma Aldrich.

### 2.2. Preparation of CNMC

The bimetallic Cu-Ni nanoparticles coated mesoporous carbon was obtained using the same method [11]. Briefly, Cu(CH_3_COO)_2_·3H_2_O and Ni(CH_3_COO)_2_·6H_2_O were placed in 20 mL N, N-dimethylformamide (DMF). The mesoporous carbon was added to the solution and left for 24 h until a homogenous mixture was obtained. Oleic acid and oleylamine were added to the solution and heated to 180 °C for 8 h using a 75 mL sealed Teflon-lined stainless autoclave. The acquired product was centrifuged at 10,000 rpm for 30 min and washed several times using ethanol and deionized water. Subsequently, the dried powder is heated up to 700 °C for 2 h at a heating rate of 5 °C per minute under an argon gas flow to obtain the final product of the mixed Cu-Ni nanoalloy decorated the mesoporous carbon (CuNi-MC).

### 2.3. Fabrication of Electrode and Sweat Solution

The catalyst ink was synthesized by adding 2 mL of ethanol to a mixture of 10 mg Ni/Cu-mesoporous composite, 2 mg GOx, and 40 μL nafion. The mixture was kept for sonication in an ice bath for 1 h. After that, 10 μL of the suspension was dropped on the GC electrode’s active surface, which was left to dry at room temperature for 1 h to obtain a uniform thin coating on the surface. The artificial sweat solution was synthesized using 5.5 mM lactic acid, 22 mM urea, 10 mM KCl, and 25 mM uric acid [13].

### 2.4. Characterization Techniques

The TEM analysis of the as-prepared catalyst was obtained by dispersing catalyst in IPA and a drop was left to dry on molybdenum grid to avoid interference with sample elements. A FEI-Thermo scientific-TALOS A FEG transmission electron microscope with a 200 kV beam voltage was used. Elemental analysis and mapping was performed using a FEI-Thermo scientific-SuperX EDS detector. The XRD analysis was carried using Rigaku, Miniflex2 Desktop, Tokyo, Japan, equipped with Cu K_α_ radiations. The and phase study structural of the as-synthesized catalyst was carried out at a scanning step of 0.02° in the 2θ range from 10° to 90°. Gamry-potentiostat/galvanostat (Ref. 600) was employed to perform the cyclic voltammetry (CV) and chronoamperometry experiments using a three-electrode cell configuration consisting of Ag/AgCl electrode as a reference electrode, the GC as the working electrode, and platinum wire as a counter electrode. The cyclic voltammetry was conducted at a potential range from −0.1 V to 0.8 V vs. Ag/AgCl at 25 mV/s, and the chronoamperometry was done at an applied potential of 0.6 V.

## 3. Results and Discussion

### 3.1. XRD and TEM

**Figure 1:**
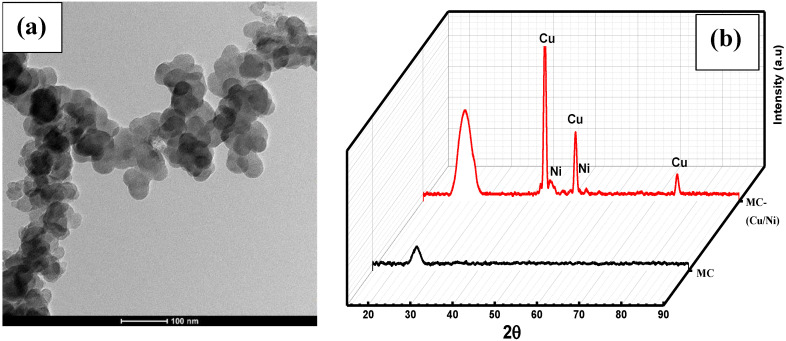
(**a**) TEM image of the used mesoporous carbon for catalyst synthesis and (**b**) XRD of bimetallic CuNi nanoparticle-coated mesoporous carbon.

Figure 1a shows the TEM image of the used mesoporous carbon synthesizing the CuNi-MC catalyst with less than 100 nm particle size. The XRD pattern of CuNi-MC is presented in Figure 1b. The average crystal size was estimated from the XRD patterns using the Debye–Scherer equation.

(1) $$D=\frac{0.9\lambda }{\beta \cos\theta }$$

where D is the crystallite size, λ is the wavelength of Cu-k_α1_ = 1.54060 Å, β can be estimated from the experimental peak width (FWHM) of the most intense peak (002). The average crystal size of the bimetallic Ni-Cu-nanoparticles was calculated in the range of 10 ± 2 nm. The amorphous peak positioned at 24.8° was attributed to the mesoporous carbon. However, the peaks located at 43.9°, 51.1°, and 75.1° are related to d values of 2.06, 1.78, and 1.26 Å, which are accredited to the well-determined (111), (200), and (220) crystallographic planes of the bimetallic Cu-Ni nanoparticles, respectively [23,44]. 

**Figure 2:**
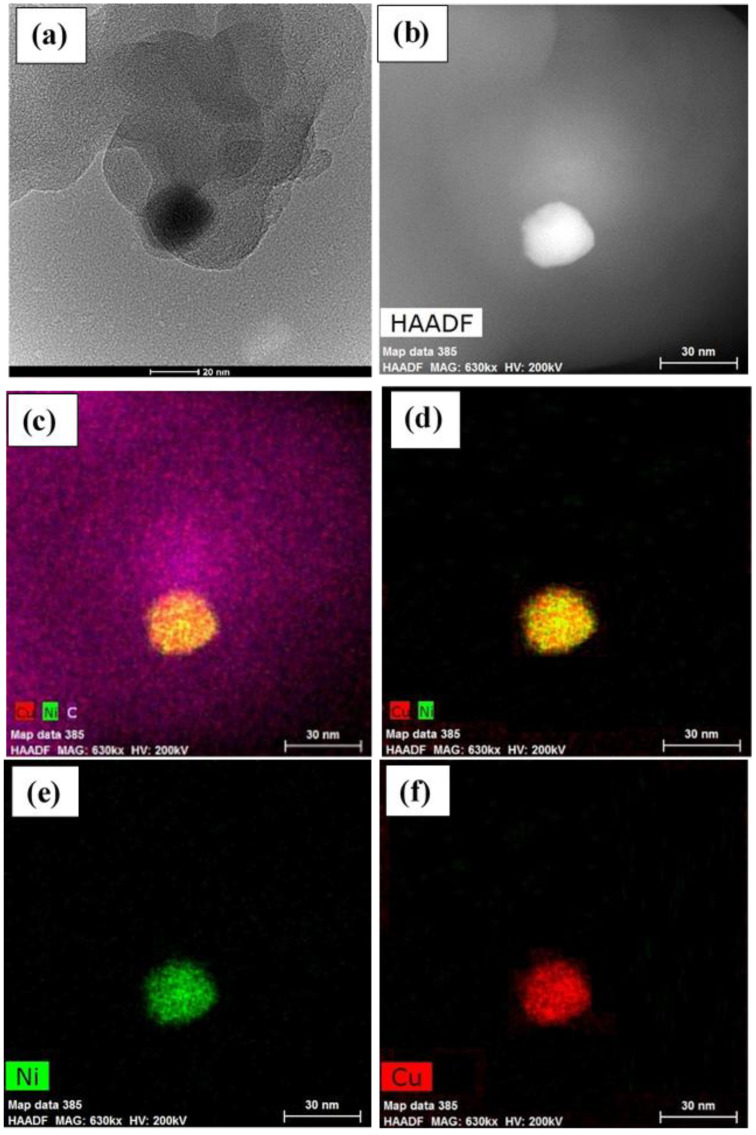
(**a**) TEM BF image of the CuNi-MC and (**b**) High-angle annular dark-field (HAADF)-STEM image, (**c**–**f**) shows elemental mapping images of bimetallic CuNi nanoparticle-coated mesoporous carbon.

The morphological studies of the as-prepared catalyst were explored using TEM (Figure 2a). The bimetallic Cu/Ni nanoparticles have a spherical shape attached to the mesoporous carbon surface with an average size of 22 ± 5 nm. The STEM and elemental mapping analysis illustrated the construction of mixed Cu/Ni nanoalloy, see Figure 2b–f.

### 3.2. Electrochemical Studies

**Figure 3:**
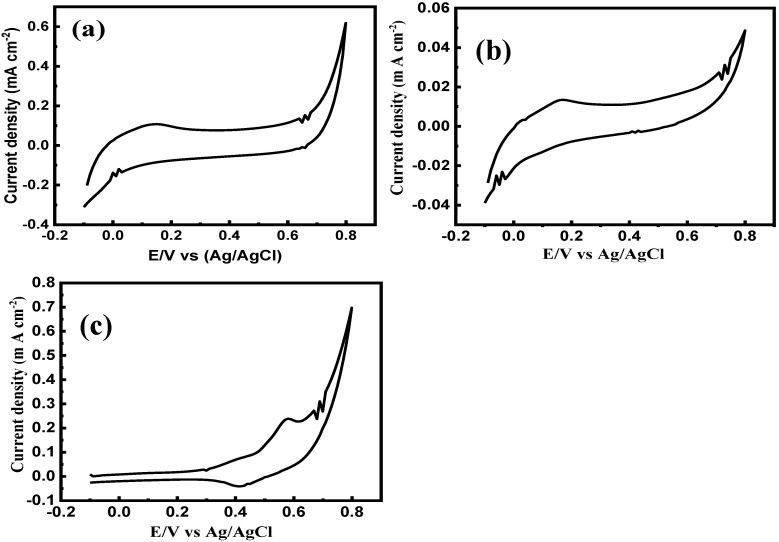
(**a**) CV of the enzymatic CuNi-MC in sweat solution after adding glucose of 0.11 mM at pH 7. The CV of CuNi-MC in sweat solution (**b**) before (**c**) after the addition of the glucose oxidase (0.11 mM) at pH 12. For all measurements, a scan rate was 50 mV s^−1^.

The glucose sensitivity of the as-prepared catalyst was evaluated at variable pH of 7 and 12. Figure 3a shows the CV of the glucose sensor at pH 7, exhibiting no oxidation-reduction peaks. Similar studies have been carried out exploring the effect of the pH on the electrode sensitivity, revealing that the catalyst sensitivity to glucose is boosted by increasing the pH [45,46,47]. The reduced species transferred the excess electrons to the Ni^2+^/Ni^3+^ redox couple leading to an enhancement in the redox current. It is worth mentioning that ion contamination limitation can be eliminated employing alkaline conditions on the electrode surface, as the hydroxyl groups (OH¯) suppress the chloride (Cl¯) adsorption to the electrode surface [45,47]. Accordingly, changing the real pH of the sweat solution in this work significantly enhanced the sensitivity of the as-prepared electrode. Figure 3b,c exhibited the CV of the as-synthesized catalyst in the absence and presence of glucose oxidase, showing a distinct variation in their response to glucose. This was ascribed to the glucose oxidation by GOx enzyme-generating H_2_O_2_, which was reduced to H_2_O + O_2_ at CuNi-MC surface and consequently increased the electrocatalytic currents.

**Figure 4:**
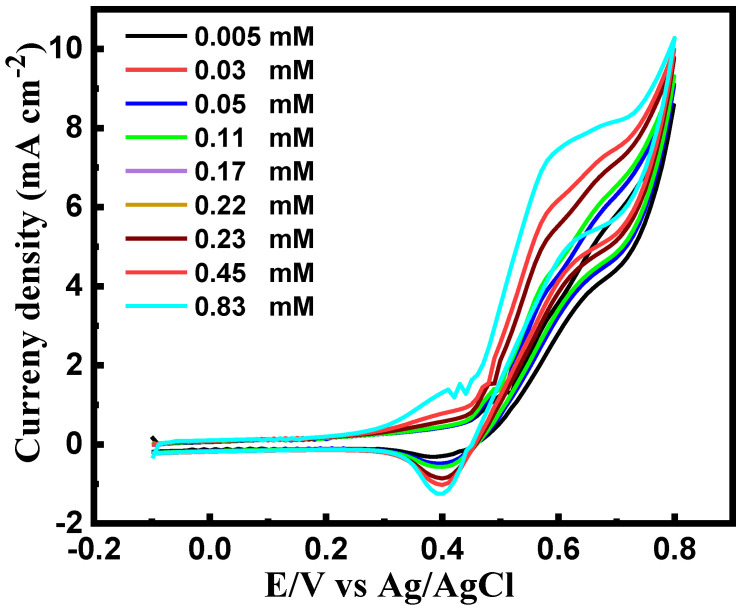
The cyclic voltammetric response of Cu-Ni modified electrode on sweat solution at pH 12 and a scan rate of 50 mV s^−1^.

Figure 4 shows the CV of the electrode modified with CuNi-MC in sweat solution at different concentrations of glucose. The redox peak potential of CuNi-MC could be linked to the redox couple of Ni^2+^ to Ni^3+^ and Cu^2+^ to Cu^3+^ and then back to Ni^2+^ and Cu^2+^. This finding indicates that the direct electrochemical reaction of GO_x_ occurs on the CuNi-MC/GO_x_ electrode surface, with GO_x_ maintaining its electroactivity on the CuNi-MC/GO_x_ surface. The current density increased by adding a higher amount of glucose, which could be accredited to the existence of many active sites due to the porous structure of the mesoporous carbon resulting in a high electron transfer rate. A Ni(OH)_2_ (Equation (5)) film is first constructed on the bimetallic CuNi surface, leading to Cu enrichment on the metal surface followed by oxidation to CuO and Cu(OH)_2_ (Equations (2) and (3)). Consequently, the film surface is composed of Cu(OH)_2_ and NiOOH, providing high electrical conductivity for the reaction [48]. Moreover, some CuO and Cu(OH)_2_ can be further oxidized to Cu^3+^ oxide [49]. Mathew et al. [50] demonstrated that an electroactive species of Ni(OH)_2_ could be reconstructed with a predominant α-structure during the reaction of γ-NiO(OH) with glucose while testing their enzymatic nickel hydroxide sensor in an alkaline medium. The generated α-Ni(OH)_2_ will be oxidized to γ-NiO(OH) during the redox reaction, boosting the anodic current. Consequently, concentrated Ni^3+^ will be formed on the surface and reduced to Ni^2+^ during the glucose oxidation reaction, increasing the cathodic current. It is worth mentioning that few of γ-NiO(OH) species could be converted to β-Ni(OH)_2_, generating NiH or NiO as an irreversible reaction since the test was carried out in an alkaline medium where α-Ni(OH)_2_ was not stable [50]. Nonetheless, the presence of higher content of Ni^2+^ and Ni^3+^ ions on the surface, smaller conversions could slightly influence the redox reaction and current [50]. Nevertheless, the bimetallic CuNi nanoparticles can efficiently restrain the unfavorable γ-NiOOH species construction, stabilizing β-NiOOH forms at pH 12 [51]. The presence of β-NiOOH species is considered to be an improved electroactive phase for superior electrochemical performance in an alkaline medium [52]. The presence of the CuNi bimetallic nanospecies facilitates the e¯ transfer between the GC surface and the GO_x_, improving glucose detection.

Cu+ 2OH^−^ → CuO + H_2_O + 2e^−^ (2)

CuO + H_2_O → Cu(OH)_2_ (3)

Cu(OH)_2_ + 2OH^−^ → CuOOH + H_2_O + 2e^−^ (4)

Ni+ 2OH^−^ → Ni(OH)_2_ + 2e^−^ (5)

Ni(OH)_2_ + 2OH^−^ → NiOOH + H_2_O+ 2e^−^ (6)

**Scheme 1:**
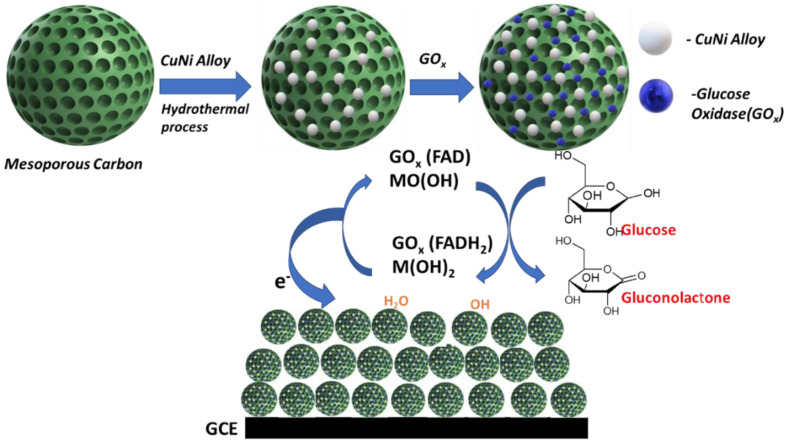
Schematic illustration for glucose sensing mechanism based on CuNi-MC/GO_x_ catalyst.

Scheme 1 describes the glucose-sensing mechanism of the enzymatic biosensor. GOx catalyzes the glucose oxidation to gluconolactone in the existence of oxygen while generating (H_2_O_2_) hydrogen peroxide and water as by-products. In addition, further carboxylic acid, gluconic acid are formed as a result of the reaction of gluconolactone and water. On the other hand, (FAD^+^) flavin adenine dinucleotide is employed as a redox cofactor for GOx to perform this oxidation reaction. FAD^+^ acts as an electron acceptor, which will be reduced to FADH_2_ during the redox process [53]. Subsequent reaction with (O_2_) to yield hydrogen peroxide revive the FAD^+^ cofactor. This process takes place at the anode surface, where the number of transferred electrons can be interrelated to the quantity of generated H_2_O_2_ and, therefore, the glucose content [53].

GO_x_(FAD) + Glucose → GO_x_(FADH_2_) + Gluconolactone (7)

GO_x_(FADH_2_) → GO_x_(FAD) + 2e^−^ + 2H^+^ (8)

NiOOH + Glucose → Ni(OH)_2_ + Gluconolactone (9)

CuOOH + Glucose → Cu(OH) + Gluconolactone(10)

**Figure 5:**
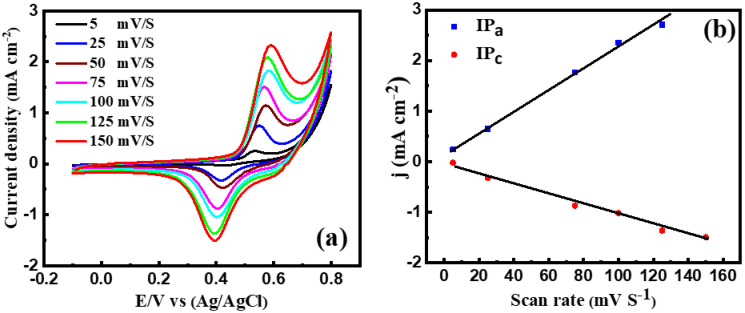
(**a**) Cyclic voltammograms (CVs) of CuNi-MC at different scan rates and (**b**) peak current density as a function of scan rates at pH 12.

The electrochemical behavior of CuNi-MC was explored at a scan rate that varied from 5 to 150 mV/s in sweat solution (Figure 5a). It is noteworthy that by increasing the scan rate, the hydroxide ions (OH^−^) penetrate the generated Ni(OH)_2_ layer giving rise to thickening the NiOOH wrought layer [54]. Both cathodic and anodic peak currents increased in proportion to the scan rates with a linear regression formula of i_pa_ = 0.186 − 0.02 v^1/2^ (n = 10, R = 0.9967) and i_pc_ = −0.034 + 0.019 v^1/2^ (n = 10, R = 0.9943), see Figure 5b. These outcomes specify that the electrochemical process of Ni(OH)_2_/NiOOH transformation is diffusion-controlled [54,55]. The i_pc_ shifted to higher negative values, and the i_pa_ shifted positively by increasing the scan rates, leading to a larger peak-to-peak potential separation. The response of CuNi nanoalloys to glucose electro-oxidation is considerably better than that of pure Cu or Ni due to the synergistic electrochemical influence of the bimetallic nanoparticles since the unalloyed Ni is not robust under highly oxidizing conditions, and Cu has a stabilizing influence on the Ni surface [56].

### 3.3. Chronoamperometric Studies

**Figure 6:**
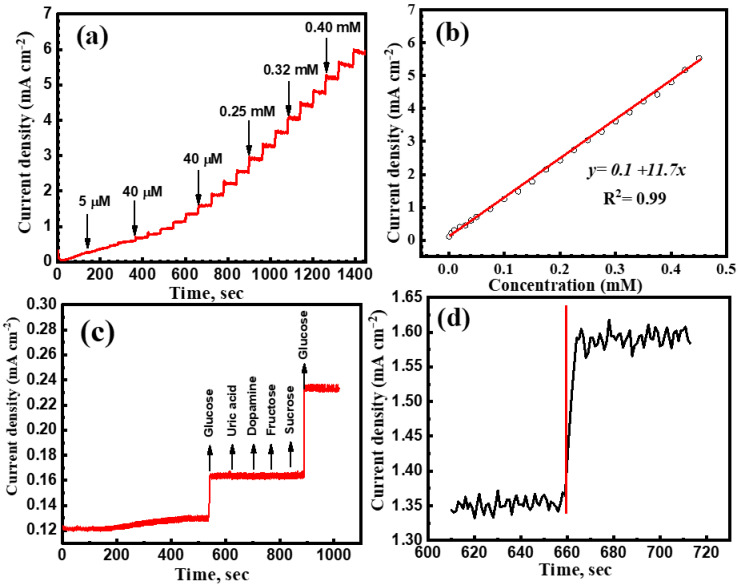
The chronoamperometric response of the mesoporous carbon coated with CuNi-MC sensor to (**a**) successive additions of higher glucose concentration, (**b**) calibration curve including the linear range, (**c**) after the addition of 40 µM glucose, uric acid, dopamine, fructose, and sucrose at pH 12 and −0.6 V (Ag/AgCl) and (**d**) response time of the sensor to reach steady-state current density after the addition of 40 µM glucose.

Figure 6a shows the corresponding amperometric response for the CuNi-MC/GOx based glucose sensor. With increasing the glucose content from 5 μm to 0.45 mM, the response of the modified electrode was enhanced due to the enhanced conductivity of the catalyst. Figure 6b exhibits a calibration plot between glucose and the current response of CuNi-MC/GOx modified electrode. The sensor showed a sensitivity of 11.7 ± 0.061 mA mM cm^−2^ with a linear range of 0.005 to 0.45 mM and a response time of 4 s. This can be explained by the high electrocatalytic activity of the as-prepared catalyst, which acquires the uniformity of the glucose oxidase (GOx) enzyme to perform the electrochemical reaction between the electrode and glucose analyte. Some easily oxidizable compounds such as fructose, sucrose, dopamine, and uric acid coexist with glucose in human sweat. The selectivity of these compounds was analyzed with amperometric measurements concerning glucose concentration. There is no substantial interference with uric acid, dopamine, fructose, and sucrose (Figure 6c). The molecular size of mesoporous carbon is 50 nm, whereas the molecular size of glucose is 0.9 nm. This might be the reason for enhancement in glucose sensitivity, as the glucose can be entrapped in the sites of the mesoporous carbon and interact with GOx enzyme with an increased surface area. Additionally, the molecular size of the other interacting species such as uric acid, dopamine, fructose, and sucrose is more or less similar to glucose. Nevertheless, the presence of GOx enzyme enhances the selectivity of glucose by interacting with the glucose molecules. Therefore, the mesoporous carbon coated on Cu-Ni is selective towards glucose rather than interfering species.

The limit of detection (LOD = 5.2 µm L^−1^) is calculated employing the following formula.

(11) $$\mathrm{LOD}=\frac{3\sigma }{m}$$

where σ and m correspond to the standard deviation of the linear range produced after the addition of glucose and the slope of the fitted calibration curve, respectively.

Figure 6d displayed that a fast current response was attained upon adding 40 µM glucose, reaching the steady-state current in less than 4 s. The rapid response time exhibits competent activity of the as-prepared catalyst toward glucose sensing.

**Figure 7:**
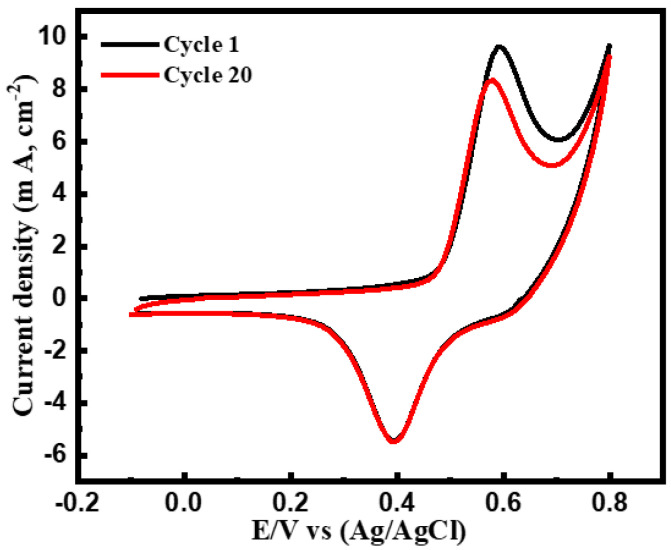
Cyclic voltammetric response of CuNi-MC electrode in 1 mM glucose solution at the first cycle and after 20 cycles at a scan rate of 50 mV s^−1^.

The stability of the as-prepared catalyst has been assessed by recording the current response of the redox couple in 1 mM glucose solution after 20 consecutive voltammetric cycles, see Figure 7. It is worth mentioning that the redox current response has insignificantly reduced after a continuous scan of 20 cycles, demonstrating that the as-prepared sensor is robust and stable.

Table 1 shows a comparative study of the mesoporous carbon’s sensitivity with Ni-Cu on glucose with different modified electrodes. The electrocatalytic activity with glucose for the electrode is depicted in terms of the electrode’s high sensitivity. We introduced the mesoporous carbon along with CuNi, which enhanced the glucose sensitivity as the surface area for glucose interaction increased. The highly conductive mesoporous carbon increases the electron transport, which results in high sensitivity of glucose. The electrode modified with mesoporous carbon coated with Ni-Cu shows high sensitivity compared to other electrodes considered here.

## 4. Conclusions

The composite mesoporous carbon coated with Ni-Cu nanoparticles can be used to fabricate wearable sensors to detect the amount of glucose in sweat. The as-synthesized catalyst’s structural and morphological properties were assessed using XRD and TEM techniques. The electrochemical analysis showed a high electroactivity of the as-synthesized catalyst to glucose electro-oxidation. The catalyst exhibited glucose sensitivity at a wide linear range from 0.005 to 0.45 mM, a low detection limit of 5.2 µM (S/N = 3), high sensitivity of 11.7 mA mM cm^−2^, and a fast response time of 4 s. The fabricated electrode’s sensitivity range was compared with other previously established electrodes suggesting that the composite is a promising material for the fabrication of wearable sensors for glucose detection. The high sensitivity and selectivity of the glucose sensor can be explained on the basis of the molecular structure and its integrations.

## Figures and Tables

**Table 1:** Comparative study of the as-prepared electrode with enzymatic electrochemical glucose sensors.

Sensor	Sensitivity (µA mM cm^−2^)	Linear Range	LOD (µm/L)	Ref.
CuNi/MC/GO_x_/Nafion/GCE	11.7 × 10^3^	5–450 μM	5.2	Current work
Au-NiO/Ni(OH)_2_-GOx	1.95	up to 30 mM	1.54 × 10^3^	[57]
GOx/Cu-hemin	22.77	9.1 μM–36 mM	2.73	[58]
MOFs				
GOx/Au-ZnO/	1.409	1–20 mM	20	[59]
GCE				
PtNWA/AuNP/	184	15 μM–2.5 mM	15	[60]
GOx				
GOx/CoS-MWCNTs/Nafion/GCE	14.9 × 10^3^	0.008–1.5 mM	5	[61]
